# Prevalence and Risk Factors of Work-Related Lower Back Pain among Radiographers in the State of Kuwait

**DOI:** 10.1155/2021/5365260

**Published:** 2021-12-03

**Authors:** Hesham N. Alrowayeh, Musaed Z. Alnaser, Talal A. Alshatti, Raed S. Saeed

**Affiliations:** ^1^Kuwait University, Faculty of Allied Health Sciences, Physical Therapy Department, Kuwait City, Kuwait; ^2^Kuwait University, Faculty of Allied Health Sciences, Occupational Therapy Department, Kuwait City, Kuwait; ^3^Kuwait University, Faculty of Allied Health Sciences, Radiological Science Department, Kuwait City, Kuwait

## Abstract

**Background:**

Work-related lower back pain (WrLBP) is a global health issue and a rising concern in the State of Kuwait. The prevalence and the risk factors of WrLBP among radiographers are not well documented.

**Objective:**

The purpose of the study was to determine the one-year prevalence, characteristics, impact, and physical risk factors of WrLBP among radiographers in the State of Kuwait.

**Methods:**

A self-administered questionnaire was distributed to 200 radiographers. The questionnaire collected data on demographics, physical risk factors, and the occurrence of WrLBP in the previous 12 months. Descriptive statistics, frequency calculations, and chi-square analyses were performed.

**Results:**

One hundred forty-six radiographers completed and returned the questionnaires with a response rate of 73% (146/200). The one-year prevalence of WrLBP was 16%. The prevalence of WrLBP was not significantly associated with the participants' demographics. Although WrLBP was significantly associated with work demands, the overall impact of WrLBP on work duties was minimal.

**Conclusions:**

The occurrence of WrLBP among radiographers in Kuwait was low, particularly when compared to healthcare providers involved in more patient handling and direct contact. However, various physical risk factors were identified. Further research is needed to investigate the effect of a treatment and prevention program on the prevalence of WrLBP.

## 1. Introduction

Healthcare professionals are often at risk of sustaining work-related musculoskeletal disorders (WMSDs) during the performance of work duties. The National Institute for Occupational Safety and Health [1] has defined WMSD as “an injury of the muscles, tendons, ligaments, nerves, joints, cartilage, bones, or blood vessels in the arms, legs, head, neck, or back that is caused or aggravated by work tasks such as lifting, pushing, and pulling. Symptoms include pain, stiffness, swelling, numbness, and tingling.” The lower back area is reported to be the most common location for WMSDs among healthcare professionals [2–4].

Several studies have investigated the prevalence of work-related lower back pain (WrLBP) among radiographers worldwide [5–8]. The 12-month WrLBP prevalence ranged from 59.6% to 83% [5–7]. Various physical and psychosocial work-related risk factors have been suggested [6–8]. Psychosocial risk factors for the development of lower back injuries among radiographers remain controversial, whereas physical risk factors have been well established [6–8].

Few studies have investigated the prevalence of WrLBP among healthcare professionals in the State of Kuwait [3, 9–11]. Of these, only one study specified the lifetime prevalence of WrLBP and possible risk factors among a small group of radiographers [9]. The lifetime prevalence was 25.7%, and transferring and lifting patients were identified as predisposing physical risk factors in the development of WrLBP.

Radiographers are mainly involved in the positioning of patients and equipment [12] in order to get the clearest and most accurate image possible. They are rarely involved in transferring and lifting patients. Thus, the study by Landry et al. [9] most probably overestimated the prevalence and misrepresented the physical risk factors (transferring or lifting patients) among radiographers in the State of Kuwait. Also, new advances in imaging technology should help to further reduce the physical workload and result in a lower prevalence of WrLPB. Furthermore, approximately 1,000 radiographers are currently working in the State of Kuwait [13]. Landry et al. [9], however, surveyed only a small proportion of them. In fact, the exact number of radiographers who participated in the study was not specified as radiographers were included in the “other healthcare professionals” category. This category also included physical therapists, laboratory technicians, pharmacists, and analgesic technicians. This may have biased the results and reduced generalizability. Thus, we aimed to examine WrLBP in a large group of radiographers working in the State of Kuwait and to report prevalence, characteristics, overall impact, and physical risk factors. The results of this study will contribute to the development of prevention and intervention strategies.

## 2. Methods

### 2.1. Participants

Radiographers of all nationalities who practiced in public or private hospitals in the State of Kuwait were randomly invited to participate in this study. Power analysis was performed, and 140 participants were deemed enough. All participants read and signed the informed consent form approved by the Ethical Review Board of Kuwait University.

### 2.2. Instrument

A three-part, self-administered questionnaire was used in this study. Part one collected information regarding the participants including age, gender, level of education, family history, exercise habits, and current work situation (i.e., duration of employment, work settings, and professional rank). Part two assessed the occurrence of WrLBP during the preceding 12 months using the Nordic questionnaire [14] and severity of the worst episode of WrLPB using the visual analogue scale (VAS). Part three assessed the physical risk factors associated with WrLBP and work demands. Participants rated how often they perform certain activities (sitting, standing, and so on) in their current job using 4-point scale varying from never to always. Excessive work demands were rated using yes/no scale.

### 2.3. Procedures

Two hundred questionnaires were distributed by a radiographer (fourth author) to prospective participants. The questionnaire was explained to each participant, and the radiographer provided a contact phone number in case further explanation should be required. Completed questionnaires were collected by the same radiographer after one week. The study protocol was approved by the Ethical Review Board of Kuwait University.

### 2.4. Data Analyses

Descriptive statistics were used to estimate the prevalence of WrLBP and analyze demographic variables. Frequencies, cross-tabulations, and chi-square tests were used to compare WrLBP prevalence by demographic variable (gender, age, and so on), work history (experience, setting, specialty, and so on), and physical risk factors. Statistical significance was set at *P* < 0.05.

## 3. Results

### 3.1. Participation Rate

One hundred forty-six radiographers completed the questionnaire; the response rate was 73% (146/200). No question was missing more than 5% of the responses. The time taken to complete the questionnaire was 5 to 10 minutes.

### 3.2. Participant Characteristics

Data regarding questionnaire responses are shown in Table 1. A higher proportion of male than female radiographers participated in this study. The mean age of participants was 37.8 ± 9.7 years (range, 23–66 years). Approximately two-thirds of radiographers were middle-aged (between 20 and 40 years). The majority of respondents (79%) were not of Kuwaiti nationality. Over 60% of participants (64%) had more than 10 years of clinical experience (mean of 15.0 ± 9.5 years). Almost all of the radiographers worked in public hospitals (95%), and most worked at least 40 hours per week (87%); the mean number of working hours was 45.7 ± 13.2 hours. Almost 39% of participants were ranked as radiographer practitioners (RS), and 52% of the participants held entry-level degrees or higher (i.e., BSc, MSc, or PhD).

### 3.3. Prevalence and Characteristics of WrLBP

The one-year prevalence of WrLBP among radiographers in this study was 16% (24/146). Almost half of the affected radiographers experienced only one episode of WrLBP (47%), and most cases lasted one to seven days (57%; Table 2). Stiffness and pain, which developed gradually, were the most common symptoms. The mean severity of the worst episode of WrLBP experienced over the past 12 months was 5.13 out of 10 using the VAS. Radiographers were mostly seen by a general practitioner, and the majority received treatment with aspirin or ibuprofen.

The prevalence of work-related lower back complaints was significantly associated with work demands (*p*=0.002; Table 3). It was however not significantly associated with gender, age, area of specialty, professional experience, workings hours, exercise, or co-worker or supervisor support (Table 3).

### 3.4. Work Activity and the Prevalence of WrLBP

WrLBP was significantly associated with the following work activities: standing, sitting, walking for prolonged periods, working with one's hands above shoulder height, reaching, lifting less than 5 kg, pushing and pulling loads, bending and/or twisting the upper body (*p* < 0.05), and squatting (*p* < 0.005) (Table 4). WrLBP was, however, not significantly associated with working posture (*p*=0.15) or with slipping or falling during the transport of loads (*p*=0.16) (Table 4).

### 3.5. Impact of LBP on Work

**Figure 1:**
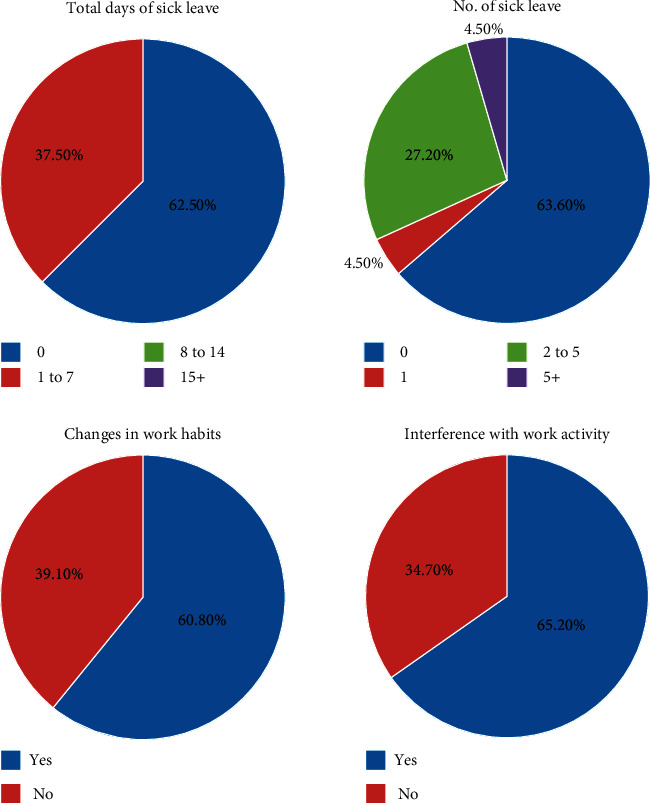
Impact of LBP on radiographers working in Kuwait. Percentage/number values do not always add up to the total percentage/number of questionnaire respondents due to missing data.

Figure 1 shows the impact of LBP on work. Absence from work due to WrLBP ranged from one to seven days. The majority of radiographers (63.6%, 14/22) who complained of WrLBP did not take any sick leave. Two-thirds of radiographers (65.2%, 15/23) were aware that WrLBP can interfere with daily work activities. Thus, most radiographers (60.8%, 14/23) made changes in their work habits such as changing their area of practice or amount of patient contact.

## 4. Discussion

We report three key findings in our study. First, WrLBP was not common among radiographers working in the State of Kuwait. Second, WrLBP was significantly associated with work demands but not with demographics, work settings, exercise habits, or co-worker or supervisor support. Third, work duties were not affected by WrLBP.

### 4.1. Prevalence Rate and Characteristics of LBP

The one-year prevalence rate of WrLBP reported by radiographers in our study (16%) was lower than most rates reported worldwide. Studies from Italy, the United States of America (USA), the Netherlands, and Canada have reported prevalence rates of almost 60% [7], 72% to 77% [5], 76% [8], and 83% [6], respectively. The disparity between our results and these results might be due to the widespread availability of radiographer assistants in Kuwait. A lower prevalence of WrLBP has previously been reported among physical therapists in Kuwait compared to those around the world as a result of the abundant help available to them [3]. Work-related physical load is significantly associated with the occurrence of musculoskeletal injuries [2], and the availability of assistants helps to reduce the physical load that radiographers must bear.

The one-year prevalence rate of WrLBP among radiographers in the State of Kuwait was lower than that reported among local physical and occupational therapists [3, 10, 11, 15]. It was also lower than that reported among physical therapists [16–21], occupational therapists [20, 21], and nurses [22–24] around the world. The high prevalence of WrLBP among these health professionals could be associated with patient handling such as transferring and lifting. As the work duties of radiographers require minimal patient handling, the prevalence of WrLBP was very low in this professional group. This finding clearly supports the notion that an increase in patient handling is associated with a growing risk of injury. The prevalence of WrLBP among radiographers in the State of Kuwait was lower also than the prevalence rate among dentists [2, 25, 26]. The lack of patient handling, however, cannot explain this difference as dental practice does not involve lifting or transferring patients. One possible explanation for the difference might be the length of time spent in static postures during work activities [26]. Ratzon et al. [26] found a significant association between length of time spent sustaining an activity and WrLBP.

### 4.2. Demographics, Exercise Habits, Co-Worker or Supervisor Support, Work Demands, and Work Settings

Age is a potential risk factor for the development of WrLBP among physical therapists and nurses [3, 24]. Age, however, as a risk factor for the development of WrLBP among radiographers is controversial [5, 7]. We found no significant association between age and the prevalence of WrLBP. This finding is in agreement with the results reported by Wright and Witt [5] but differs from those reported by Lorusso et al. [7].

In this study, there was no significant association between the prevalence of WrLBP and gender. This finding is in agreement with the results reported by Lorusso et al. [7] among radiographers. However, they contradict the findings reported among other healthcare professionals [3, 24]. These studies have shown that female healthcare professionals are more at risk of sustaining musculoskeletal injuries than males [3, 24] as a result of anatomical and physiological differences [3].

In accordance with the finding reported by Landry et al. [9], we found no significant association between exercise and the prevalence of WrLBP among radiographers in this study. This was unexpected as physical activity promotes and maintains health and fitness and should therefore reduce the risk of injury. This suggests that physical activity is not sufficient to prevent WrLBP where static and awkward postures are involved in the performance of work duties.

WrLBP among radiographers in this study was not significantly associated with co-worker or supervisor support. This was similar to the results of Bos et al. [8]. However, since the perception of one's work or meaning of work is subjective and can differ greatly from one individual to another, the association between psychosocial factors such as support and WrLBP might be inconsistently reported.

Previous studies [7, 27] have reported a positive association between length of employment and WrLBP. However, the results of this study were not in agreement. The specialty area is a potential risk factor in the development of work-related musculoskeletal pain. However, the nonsignificant association between area of specialty and prevalence of WrLBP reported in this study contradicts the findings previously reported among nurses [24], physical therapists [19], and dental office personnel [28]. A possible explanation is that areas of practice in radiography do not vary significantly in work activities and tasks, and in turn no change in the rate of prevalence of WrLBP is observed. Finally, as far as we are aware, there is a lack of studies regarding the association between working hours and prevalence of WrLBP among radiographers; we report a nonsignificant association.

### 4.3. Work Activity and the Occurrences of WrLBP

Like most studies conducted among healthcare professionals, we found that work-related physical activities were significantly associated with the occurrence of WrLBP. In this study, standing, sitting, or walking for prolonged periods of time increased the prevalence of WrLBP, indicating that cumulative physical burdens increase the prevalence of WrLBP among radiographers.

Common activities in radiological units include helping to wheel and position patients, moving X-ray machines, carrying cassettes, pushing mobile units, and moving buck trays. These duties involve squatting, static posture, working with one's hands above shoulder height, reaching far, pushing, and bending and/or twisting. These activities increase the physical load on the lower back area and intensify muscular contractions. They were significantly associated with the prevalence of WrLBP among radiographers in the present study. Therefore, using ergonomic procedures to position patients and handle equipment during clinical practice may reduce the prevalence of WrLBP. This possibility is the subject of ongoing research.

### 4.4. LBP Effect on Work

The overall effect of LBP on work was minimal. The majority of radiographers in this study did not take any sick leave from work as a result of their WrLBP. The total number of days of work lost due to LBP was 56. This is very low compared to the number of lost workdays reported by nurses [24] and physical therapists [3]. Studies have reported a loss of 202 days by nurses and 75 to 219 days by physical therapists as a result of WrLBP. These professionals frequently engage in handling, lifting, and transferring patients. They spend more time in direct contact with patients than radiographers as a result of their work duties. This might explain the difference in the impact of WrLBP.

No change in the way radiographers practiced was reported despite the fact that the majority were aware that WrLBP might interfere with their daily work activities. Furthermore, the majority of respondents did not take any sick leave to rest and recover from their WrLBP but rather continued their normal working routine. The mean VAS was 5.12 for the worst pain suffered by these radiographers during an attack of WrLBP over the past 12 months. This VAS score indicates mild to moderate pain. Absence from work might increase when LBP becomes severe [29]. In addition, the majority of radiographers received analgesic drugs during attacks of WrLBP. This rendered the mild to moderate pain tolerable enough for the radiographers to work comfortably.

### 4.5. Limitations

The main limitation of this study was recall bias. The study relied on self-reported data, and participants may not have remembered all incidents of WrLBP. Furthermore, participants may have underestimated their work-related back injuries to avoid being stereotyped by their superiors or viewed negatively based on their injury history.

## 5. Conclusion and Recommendation

The prevalence of WrLBP among radiographers in the State of Kuwait was low. However, various work-related physical risk factors were identified. Further research to evaluate the effect of a treatment and prevention program on the prevalence of WrLBP is needed. Similarly, further research is needed to document the prevalence of WMSDs in all anatomical areas of the body among radiographers in the State of Kuwait.

## Figures and Tables

**Table 1:** Study participant demographics.

Gender	No.	%
Male	87	60
Female	59	40

Age		
20–30 years	33	23
31–40 years	70	49
41–50 years	24	17
51+ years	17	12

Nationality		
Kuwaiti	30	20
Egyptian	25	17
Filipino	30	20
Indian	44	30
Other	17	12

Education		
Diploma	68	48
BSc	72	50
MSc	2	1
PhD	1	1

Professional rank		
Junior RS	27	19
RS	54	39
Senior RS	36	26
RS specialist	10	7
Senior RS specialist	9	7
Superintendent RS	3	2

Professional experience		
0–10 years	49	36
11–20 years	57	42
21+ years	30	22

Working hours		
<10–19 hours	10	7
20–39 hours	9	6
40+ hours	123	87

Work settings		
General hospital	134	95
Private hospital/clinic	7	5

Areas of specialty		
General	31	22
Special	89	62
Both	24	17

Column values do not always add up to the total number of questionnaire respondents due to missing data. Others nationalities include Iranian, Jordanian, Polish, Sudanese, Syrian, and Yemeni. For areas of specialty, special includes CT, MRI, fluoroscopy, and mammography.

**Table 2:** Characteristics of LBP among radiographers working in Kuwait.

	LBP	
Number of spells	No.	%
1	9	47
2 to 5	7	37
6+	3	16

Longest spell		
1 to 7 days	13	57
2 to 3 weeks	5	22
3to 4 weeks	2	9
3+ months	3	13

Nature of complaints		
Stiffness	10	50
Nagging feeling	0	0
Numbness	2	10
Tingling	0	0
Loss of strength	1	5
Cramp and spasm	2	10
Pain	5	25

Onset of complaints		
Sudden	10	44
Gradual	13	57

Expert seen		
No visit	6	40
Physical therapist	3	20
General practitioner	4	27
A specialist	2	13

Treatment received		
PT	3	21
Drugs	6	43
No treatment	5	36

Column values do not always add up to the total number of questionnaire respondents due to missing data.

**Table 3:** Prevalence and association of LBP among radiographers working in Kuwait with demographics, work settings, exercise habits, and physical risk factors.

Gender	LBP	
	*P*	Χ^2^ value
Male	0.470	0.522
Female		

Age		
20–30	0.745	1.233
31–40		
41–50		
51+		

Area of specialty		
General	0.074	5.217
Special		
Both		

Professional experience		
1–10	0.822	0.393
11–20		
21+		

Working hours		
<10	0.985	0.149
10–19		
20–29		
30–39		
40+		

Exercise		
Yes	0.267	1.233
No		

Work demands		
Yes	0.002^*∗*^	9.473
No		

Co-worker support		
Yes	0.6	0.275
No		

Supervisor support		
Yes	0.15	1.739
No		

For area of specialty, special includes CT, MRI, fluoroscopy, and mammography. ∗ indicates statistically significant difference at *P*=0.05.

**Table 4:** Prevalence of LBP among radiographers working in Kuwait and association with physical risk factors (work activity).

Work activity	LBP	
	*P*	Χ^2^ value
Standing	0.012^*∗*^	6.255
Sitting	0.024^*∗*^	5.128
Walking	0.022^*∗*^	5.232
Squatting	0.006^*∗*^	7.657
Overhead activity	0.025^*∗*^	5.011
Reaching	0.011^*∗*^	6.487
Lifting less than 5 kg	0.020^*∗*^	5.400
Pushing/pulling	0.020^*∗*^	5.400
Slipping/falling	0.169	1.889
Bending/twisting	0.048^*∗*^	3.980
Improper body mechanism	0.150	2.069

∗ indicates statistically significant association at *P*=0.05.

## Data Availability

The data that support the findings of this study are available on request from the corresponding author. The data are not publicly available due to privacy and ethical restrictions.
